# Validation of reference genes for the normalization of the RT-qPCR gene expression of virulence genes of *Erwinia amylovora* in apple shoots

**DOI:** 10.1038/s41598-017-02078-4

**Published:** 2017-05-17

**Authors:** Monika Kałużna, Anita Kuras, Joanna Puławska

**Affiliations:** Research Institute of Horticulture, Konstytucji 3 Maja 1/3, 96-100 Skierniewice, Poland

## Abstract

To study the expression of pathogenicity-related genes in *Erwinia amylovora*, seven candidate reference genes (*ffh*, *glyA*, *gyrA*, *proC*, *pykA*, *recA*, *rpoB*) were selected and validated with the following five different mathematic algorithms: geNorm, NormFinder, BestKeeper, the delta CT method and the RefFinder web-based tool. An overall comprehensive ranking output from each of the selected software programs revealed that *proC* and *recA*, followed by *ffh* and *pykA*, were the most stably expressed genes and can be recommended for the normalization of RT-qPCR data. A combination of the three reference genes, *proC*, *recA* and *ffh*, allowed for the accurate expression analysis of *amsB* and *hrpN* genes and the calculation of their fold change in *E. amylovora* after its infection of susceptible and resistant apple cultivars. To the best of our knowledge, this is the first study presenting a list of the most suitable reference genes for use in the relative quantification of target gene expression in *E. amylovora in planta*, selected on the basis of a multi-algorithm analysis.

## Introduction

*Erwinia amylovora* is a bacterial pathogen that affects more than 130 plant species belonging to 40 genera, mainly from the family *Rosaceae*. It causes fire blight, which is the most serious bacterial disease of pome fruits. As a member of *Enterobacteriaceae*, *E. amylovora* is related to many important human and animal pathogens such as *Escherichia coli*, *Yersinia pestis*, *Yersinia enterocolitica*, *Salmonella enterica* and *Shigella flexneri*. Although the *E. amylovora* strains are known to be very homogenous in terms of phenotypic and genetic features^1^ they show significant differences in the level of virulence^2–5^. The greatest diversity in the virulence of the tested strains was observed in apple cultivars such as Quinte or Free Redstar, which are regarded as resistant to fire blight^6, 7^. The reasons for these differences are not well known or studied; however, we assume that differences in virulence between the strains of *E. amylovora* can be caused by the differential expression of pathogenicity-related genes or the presence of new, unrecognized virulence factors. Currently, several genes involved in the pathogenicity of *E. amylovora* have been described^8^. Among them, the most crucial is the *hrp* type III secretion system with a repertoire of type III effectors^9^ and the extracellular polysaccharide (EPS) amylovoran^10^. However, little is known about their expression in different strains during their infection of different hosts.

Reverse transcription quantitative real-time polymerase chain reaction (RT-qPCR)^11^, is one of the most commonly used techniques to study gene expression and validate data obtained from RNA sequencing (RNA-seq)^12–14^. Studies of gene expression can provide information about differences in gene expression between samples, providing information related to complex regulatory networks and interactions between hosts and pathogens^15^. Absolute quantification (digital PCR method/standard curve method) and relative quantification are two methods of quantitative PCR utilized in research. In the latter one, the relative expression of a target gene is determined compared to a standard reference gene, and it is the best and most common method utilized for the analysis of relative changes in the mRNA expression of a target gene^16, 17^. The success and usefulness of RT-qPCR for research can be attributed its rapidity, reportativity, high specificity and sensitivity^18, 19^. However, high-fidelity reactions and the accuracy of the results of gene expression profile analyses are affected by several factors, mainly associated with the standardization of pre-analytical steps^20^. On the one hand, the preparation and precise execution of the reaction itself includes (i) the careful examination of RNA extraction methods prior to their use to obtain high values of RNA integrity^21, 22^, which may affect the results of downstream applications including RT-qPCR, and (ii) the high efficiency obtained in the reverse transcription reaction^23, 24^. On the other hand, RT-qPCR data and obtaining credible results^11, 25, 26^ depends on the normalization process, and the proper selection of reference genes. Usually, genes that serve as internal controls for the normalization of RT-qPCR are genes of core genomic function; for example, housekeeping genes are supposed to have stable expression under different conditions. Ideal reference genes should exhibit little variation in expression in different samples independent of the experimental conditions^27, 28^. The application of unstably expressed genes for normalization will lead to inaccuracy or false results and conclusions^12, 26^. However, there is not an ideal set of genes that can be used in all organisms; therefore, it is necessary to select and analyse a set of genes suitable in each particular case for gene expression analysis prior to RT-qPCR. In searching for the appropriate reference gene set, we are supported by guidelines from one site called the Minimum Information for Publication of Quantitative Real-Time PCR Experiments (MIQE^11, 29^) and by several mathematic algorithms, which are widely applied for normalization of data^30–34^.

Currently, only a few papers concerning the selection and validation of reference genes for the normalization of RT-qPCR gene expression and for transcriptomic analyses for plant pathogenic bacteria or plants after infection with plant pathogens have been published^12, 35, 36^. Furthermore, there is no published work aimed at identifying effective reference genes for RT-qPCR using the multi-algorithm method to study gene expression in the plant pathogen *E. amylovora*.

The aim of our study was to identify the most appropriate reference genes and to quantify the expression of the pathogenicity-related genes *amsB* and *hrpN* in *E. amylovora* after its inoculation to apple tree shoots. For this study, eight candidate reference genes, *ffh*, *glyA*, *gyrA*, *proC*, *pykA*, *recA*, *rpoB* and *gyrB*, were selected based on a literature screen of commonly used housekeeping genes for RT-qPCR in other bacterial species, and different mathematic algorithms were employed to determine their suitability.

## Results

### Selection of candidate reference genes and primer design

Of the eight candidate reference genes for which primers were designed, seven (*ffh*, *glyA*, *gyrA*, *proC*, *pykA*, *recA*, *rpoB;* Table 1) were selected for further study of the stability of the gene expression and the ranking of the reference genes based on the results obtained from both PCRs. For *gyrB*, although several primer pairs were designed and single products were obtained in classical PCR, the efficacy of real-time PCR was very low. Gene names, primer sequences, amplicon lengths, melting temperatures (T_m_), PCR efficiency, and regression coefficients of the reference genes selected are given in Table 1. All the primer pairs used in this study allowed us to obtain a single product of the expected size between 107 to 168 bp. (Table 1 and Supplementary Figure S1). The melting curves of the reaction products obtained in real-time PCR revealed a single peak with a T_m_ ranging from 84 °C to 88 °C. Additionally, neither unexpected nor additional peaks in the product melting curves were observed (Supplementary Figure S2), which clearly excluded the possibility or tendency of the primers to form dimers. The calculated efficiencies for the reference genes vary from 96 to 101.6%, with linear correlation coefficients (r^2^) ranging from 0.996 to 1.00 (Table 1).

**Table 1:** Primers for reference and pathogenicity related genes of *Erwinia amylovora* for expression studies with parameters obtained from RT-qPCR.

Gene name	Primer sequence (5′-3′)	Amplicon length (bp)	Amplicon Tm (°C)	PCR efficiency (%)	Regression coefficient (r^2^)	Reference
*ffh*	Forward: GGTCGGGGTAGATTTTTGTCCTTC	153	85–85.5	99.7	0.998	This study
	Reverse: ATCTCGTCCATCATCGCTTCATC					
*glyA*	Forward: GCCTTCAGCATATTTGTTGGTCAG	114	84	100.2	0.999	This study
	Reverse: CAGGAGAAAGTGCGTCAGGAAG					
*gyrA*	Forward: TTACCGGCGGCAGAAAACAG	107	85.5	101.6	0.996	This study
	Reverse: CGCAGCGCCGGTATTATTG					
*proC*	Forward: TGCCGGCCACATCCTTCAG	151	88	100.5	1.00	This study
	Reverse: GACCATAAACCCGCCACTAATCAG					
*pykA*	Forward: CCATTCTCGGCGACCTCCAG	127	85.5	96	0.999	This study
	Reverse: TCTTTGTTGCCTTCGCTTTTACCC					
*recA*	Forward: CGATGACAACAAGCAAAAAGCACT	144	86.5	99.5	0.998	This study
	Reverse: GCGATATCCAGCGACAAAGAGC					
*rpoB*	Forward: AAGACTCTTCTCTGCGCGTA	168	85.5	100.5	0.999	This study
	Reverse: CAGCTTCGAGGATCTGCAAC					
*amsB*	Forward: GCGGTAATTTATAGGCTTTGTAGG	85	81.5	104.9	0.996	This study
	Reverse: AAGTATTCTCTGTTCTGGCTGGAC					
*hrpN*	Forward: CCTGAGCGGGCCGGTGGACTAC	146	86	95	0.998	This study
	Reverse: TCGCCCGATCGCCTTTATTGAC					

### Expression levels of the candidate reference genes

**Figure 1:**
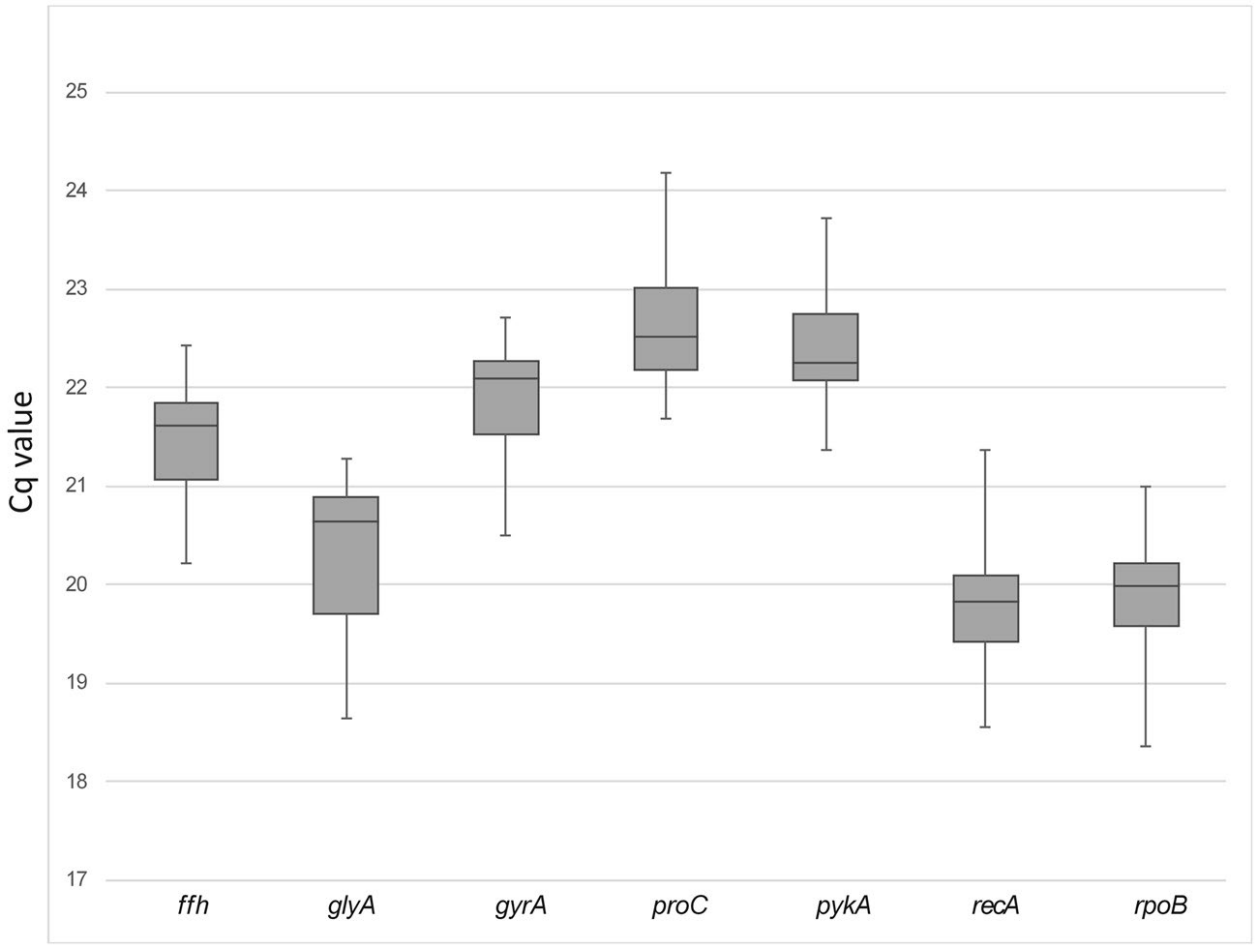
Cq values (expression levels) for seven candidate reference genes in all samples tested. The box indicates the 25th and 75th percentiles and the whiskers caps represent the maximum and minimum values. A centre line across the boxes indicate the median.

The analyses of Cq values from the seven selected reference genes showed relatively broad differences between them, indicating differential expression and showing the necessity of using a statistical method to rank the stability of these genes and determine the most accurate reference for gene expression studies (Fig. 1). The Cq values of all genes ranged from 18.36 to 24.18. The median and average Cq values were not too distant for all of the reference genes. The *recA* gene exhibited the lowest mean value (19.83), meaning that it was expressed at the highest level, while *proC* was the least expressed gene with the highest mean Cq (22.51). The Cq values for the seven reference genes evaluated in the different samples are represented in a box-and-whiskers plot (Fig. 1).

### Stability of the reference genes

The stability of expression for the seven reference genes selected was analysed using four statistical algorithms for each of the RNA samples: geNorm, NormFinder, BestKeeper and the delta-Ct method. The seven candidate reference genes were evaluated by each program from the most to the least stably expressed genes. Finally, the geometric mean (GM) of each gene was then calculated, and the genes were re-ranked using the RefFinder web-based tool. The results from all analyses are presented in Table 2.

**Table 2:** Stability values and ranking order of seven candidate reference genes obtained of all analysed samples from Idared and Free Redstar based on results from geNorm, NormFinder, BestKeeper, Delta Ct and Comprehensive ranking.

Ranking	Genorm		NormFinder		BestKeeper		Delta Ct		Comprehensive ranking	
	gene	M-value*	gene	Stability value*	gene	Std dev [ ± CP]*	gene	Average of st dev*	Gene	Geomean of ranking values (GM)*
1	*proC*	0.550	*proC*	0.077	*ffh*	0.46	*proC*	0.55	*proC*	1.41
2	*recA*	0.558	*pykA*	0.091	*pykA*	0.48	*recA*	0.56	*recA*	2.06
3	*pykA*	0.561	*recA*	0.094	*recA*	0.49	*pykA*	0.57	*ffh*	2.63
4	*ffh*	0.603	*ffh*	0.104	*proC*	0.50	*ffh*	0.60	*pykA*	2.63
5	*gyrA*	0.631	*gyrA*	0.123	*gyrA*	0.57	*gyrA*	0.63	*gyrA*	5.23
6	*rpoB*	0.661	*rpoB*	0.135	*rpoB*	0.59	*rpoB*	0.66	*rpoB*	5.73
7	*glyA*	0.747	*glyA*	0.163	*glyA*	0.67	*glyA*	0.75	*glyA*	7.00

*As lower value as more stable gene.

**Figure 2:**
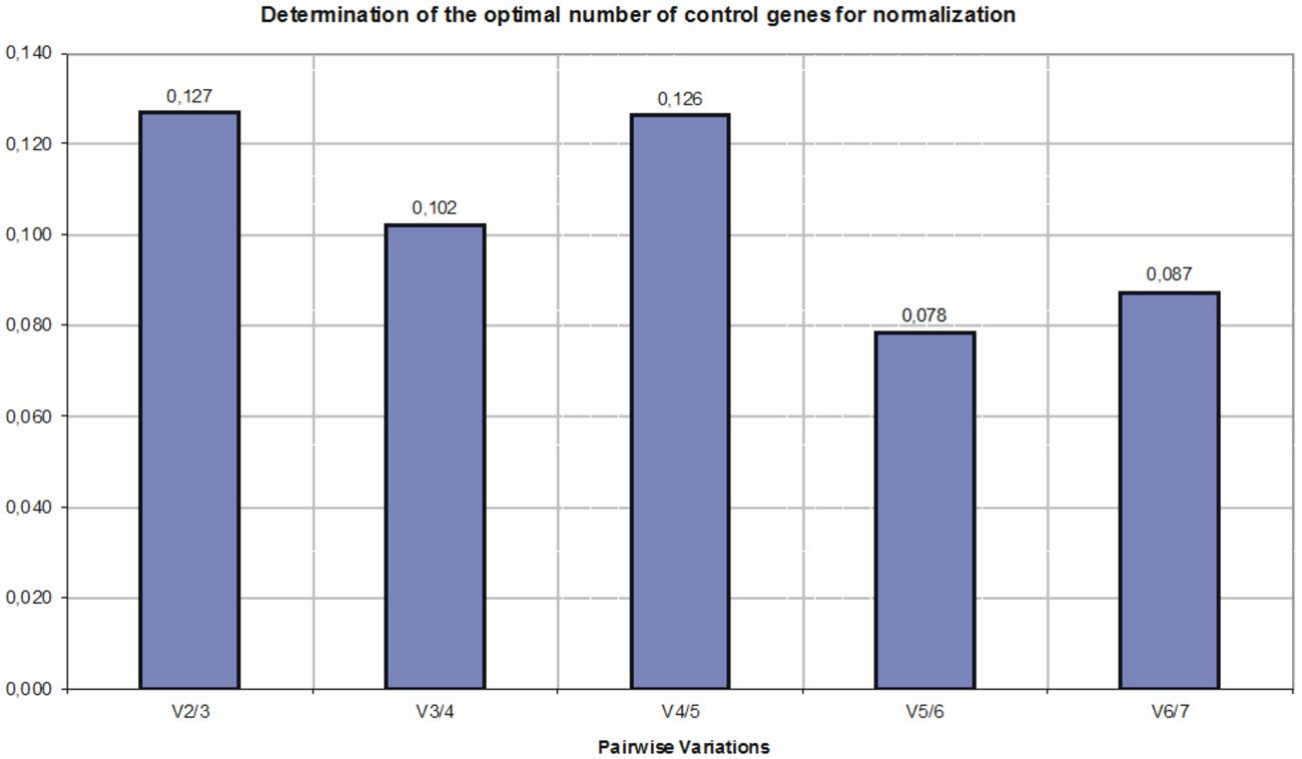
Optimal number of reference genes fo r accurate normalization calculated by geNorm analysis. Pairwise variation (V_n_/V_n + 1_) analysis is calculated between the normalization factors NF_n_ and NF_n+1_ to determine the number of control genes required for accurate qRT-PCR normalization. 0.15 is proposed as a cut-off value, below which the inclusion of an additional reference gene is not required (Vandesompele *et al*.^30^).

According to the geNorm analysis, the *proC*, *recA*, *pykA* and *ffh* genes had the lowest M-value (0.550, 0.558, 0.561, 0.603, respectively), indicating the highest stability (Table 2). The *glyA* was the least stably expressed gene. All the analysed reference genes showed an M-value below the determined default limit of M < 1.5, confirming stability in different conditions. Pairwise variation (V-value) was calculated for the reference genes to determine the minimum number of genes necessary for accurate data normalization; this analysis revealed that the pairwise variation value V2/3 was below the default threshold value of 0.15 (Fig. 2). Therefore, 2 genes can be used for normalization, and according to program assumptions, the additional inclusion of more reference genes will have no significant contribution to the normalization of the expression of the studied genes.

The Normfinder analysis showed similar results to those obtained by geNorm and revealed that the most stably expressed genes are *proC* and *pykA* (SV: 0.077 and 0.091), followed by *recA* and *ffh;* furthermore, the analysis indicated *glyA* as the least stably expressed gene, confirming the geNorm results (Table 2). The NormFinder algorithm showed that that *proC* and *recA* constitute the best combination of two genes with a stability value 0.061.

Based on the results from the BestKeeper analysis, all genes were calculated to have an SD value lower than 1. The genes *ffh* and *pykA* (SD: 0.46 and 0.48) were highlighted to be the most stably expressed, followed by *recA* and *proC* with nearly the same values (0.49 and 0.50) as most stably expressed genes (Table 2). *glyA* was the least stably expressed gene.

Using the delta Ct method, where no primer efficiency is included for the calculations, the ranking of the reference genes was mostly similar to the obtained by the geNorm algorithm (Table 2). *proC* and *recA* (SD: 0.55, 0.56) were determined to be the most stably expressed genes, followed by *pykA*, *ffh* and *gyrA* (SD: 0.57, 0.60, 0.63, respectively).

When the raw data were introduced into the RefFinder web-based tool, which adopts the same value of primer efficiency equal to 100%, the ranking of reference genes was the same as that obtained by geNorm and NormFinder, to which relative quantities, not raw data, are imported. The BestKeeper output also yielded the same data, although the efficiency value was not introduced. For the output results of the delta Ct method where untransformed raw data values constitute the input, the reference gene ranking was in agreement with the RefFinder software. An overall comprehensive ranking output revealed that *proC*, followed by *recA*, *ffh* and *pykA* (GM: 1.41, 2.06, 2.63, 2.63, respectively), were the most stably expressed genes, while *rpoB* and *glyA* were the least stably expressed (Table 2 and Supplementary Figure S3A (comprehensive ranking); Figure S3B (geNorm ranking); Figure S3C (NormFinder ranking); Figure S3D (BestKeeper ranking); Figure S3E (Delta Ct ranking).

### Expression analysis of the target genes

The expression profile analysis of the target critical virulence factors *amsB*, involved in the biosynthesis of amylovoran, and *hrpN*, encoding harpin – a secreted protein which elicits the hypersensitive response (HR) in non-hosts and is also required for pathogenicity in host plants, were selected for the study of their expression. Based on the overall comprehensive ranking the three reference genes: *proC*, *recA* and *ffh* were selected for normalization. Based on the analysis of the gene expression differences in both genes, as expected, a similar relative expression was observed–both of them were up-regulated *in planta*. However, the level of the up-regulation differed between the susceptible and resistant apple genotypes.

**Figure 3:**
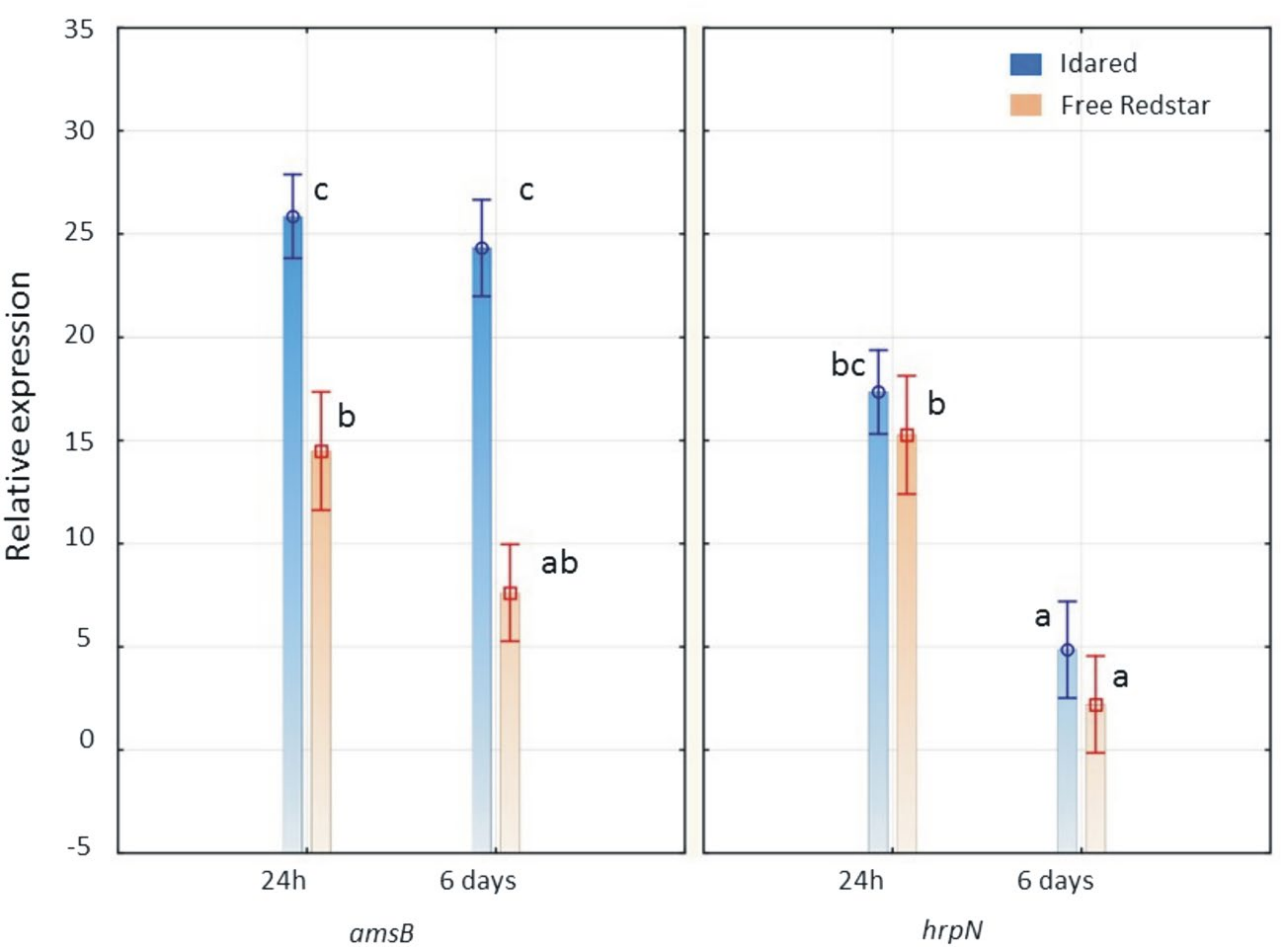
Relative expression of the *amsB* and *hrpN* genes in apple shoots, in two time points after inoculation (24 h and 6 days) in comparison to expression in pure bacterial culture, normalized with the most stable reference genes *proC*, *recA* and *ffh* selected based on different mathematical algorithms used in this study. The vertical bars represent standard error. The data that do not differ significantly from one another are marked with the same letter.

In the case of cv. Idared, *amsB* was up regulated 25.8- and 24.3-fold after 24 and 6 days after inoculation (dpi), respectively, compared to a pure bacterial culture. Therefore, the level was quite similar and was maintained during the infection process. When the resistant apple cultivar, Free Redstar, was inoculated, *amsB* was up regulated 14.4- and 7.6-fold after 24 hours and 6 days after inoculation, respectively, compared to a pure bacterial culture (Fig. 3).

For *hrpN* gene, up-regulation *in planta* compared to a pure bacterial culture was also noted. However, in both apple cultivars, a notable decrease was observed after 6 days, compared to expression after 24 h after inoculation. The expression of the *hrpN* gene was 6.91- and 3.57-fold lower after 6 days compared to 24 h after inoculation in Free Redstar and Idared cultivars, respectively. The expression level of this gene after 6 days for the Free Redstar cultivar was considered as “no change” compared to a pure bacterial culture compared to the regulation threshold (Fig. 3). The data resulting from the *proC*, *recA* and *ffh* genes as references in the relative gene expression analysis are in agreement with those obtained after the preliminary analysis of the expression of *amsB* and *hrpN* in transcriptomes obtained after RNA seq^37^.

## Discussion

The study presented allowed for the selection of stably expressed reference genes for the normalization of RT-qPCR gene expression analysis and the preliminary assessment of the expression of virulence genes in *Erwinia amylovora* after the infection of susceptible and resistant apple genotypes. Although the analysis of the gene expression of *E. amylovora* using real-time PCR was already studied^38, 39^, to the best of our knowledge, this is the first study of the validation of reference genes using a multi-algorithm analysis for use with the relative quantification of target gene expression in this bacterial species.

The study of gene expression using RT-qPCR is one of the most commonly and successfully used techniques in molecular plant pathology^12–14, 35^. The selection of an appropriate method of RNA isolation for a particular organism to obtain high quality RNA and the validation of reference genes to be used as an internal control for relative quantification is recommended for each species and for each experimental condition^34^. Recently, many papers describing the validation of reference genes in different plant species, insect or viruses have been published^12, 13, 40, 41^. However, in the case of bacteria, especially phytopathogenic bacteria, the number of publications is limited^35, 42, 43^. Moreover, in these papers, the authors indicated that there is not an ideal reference gene or set of genes that can be used for all bacteria or other plant or insect species. Therefore, determining a stably expressed set of genes for a particular species is necessary.

In the present study, eight reference housekeeping genes of *E. amylovora* were selected, and their expression was evaluated. After primer design, the first step was to evaluate the specificity and usefulness of the primers, including the determination of the amplification efficiency of each reference gene based on the conventional standard curve method. Although the alternative programs Real-time PCR Miners^44^ and LinReg PCR^45^ were described a few years ago, for the calculation of efficiency in publications concerning the validation of reference genes, the use of the standard curve method is still preferred^12, 46^.

To rank the candidate reference genes from the most to least stably expressed and to select the most useful for determining the expression of pathogenicity-related genes (*amsB* and *hrpN*), several different mathematical algorithms were adopted^12, 40, 43, 46, 47^. The comprehensive ranging obtained based on the results of all the algorithms used showed that *proC*, followed by *recA* and *ffh*, were the most stably expressed set of reference genes. The *proC* gene was also identified as one of two stably expressed genes in *Aeromonas salmonicida* subsp. *salmonicida* ^43^. The *recA* gene was found within a set of the most stably expressed reference genes validated for qPCR studies in *Staphylococcus pseudintermedius* ^48^. Among *Xanthomonas citri* subsp. *citri* genes studied during an infection of *Citrus sinensis, rpoB* was found within a group of most stably expressed genes^35^, while in our study it was one of least stably expressed genes. Similarly, as in our research, the *recA* and *ffh* genes proved to be the best reference genes studied for the normalization of reference genes for *Pectobacterium atrosepticum* ^36^. However, a different set of genes was found to be the most appropriate reference genes for *Azospirillum brasilense*. An analysis based on the three software programs indicated that in case of this species, *gyrA*, *glyA* and *recA* were the most stably expressed reference genes^49^. Therefore, the results obtained for only *recA* were in agreement to our findings. The variation in results obtained for particular species and conditions indicate that it is necessary to conduct gene normalization in each case, condition or species. On the other hand, it is worth emphasizing that all the reference genes analysed in our study showed an M-value below the determined default limit of M < 1.5, and the SD values in BestKeeper were <1, confirming adequate stability under different conditions.

It is known from previous studies that there are some discrepancies in gene ranking and validation when generated by different programs. In the majority of studies, as in ours, these small differences in gene ranking were present using BestKeeper compared to geNorm and NormFinder^12, 40, 43^. The discrepancies are connected to differences existing in the statistical algorithms used in each program^31, 34^ thus application of different algorithms for selection of reference genes is valuable.

According to the geNorm pairwise variation value (V_n/n+1_ value), minimum/optimal number of genes for accurate data normalization is 2 (V2/3 below the threshold value 0.15) (Fig. 2); therefore, 2 genes can be used for normalization, and according to the program assumptions, the inclusion of additional reference genes has no significant impact on the normalization of gene expression. On the other hand, the proposed value of 0.15 should not be considered a strict a cut-off. As stated by Vandesompele *et al*.^30^, the graph is only intended to be a guide for determining the optimal number of reference genes. Using the 3 best reference genes, as we used in our research, is a valid normalization strategy in most cases, and the results are much more accurate and reliable compared to the use of only one single reference gene^30^. According to the MIQE Guidelines, at least two reference genes to determine gene expression changes is acceptable and required; however, three reference genes is preferred, and the use of only one reference gene is not acceptable^11^, as has been shown to lead to the distortion of the results obtained^43^. In our study, three genes, *proC*, *recA* and *ffh*, were shown to be the most appropriate. We conducted a gene expression analysis of *E. amylovora* pathogenicity related genes, *amsB* and *hrpN*, and showed that these genes were highly up regulated, mostly 24 hours after inoculation; this result seemed reasonable as these genes are essential pathogenicity factors and are required for infection. The results of the gene expression analysis performed for genes involved in pathogenicity of *E. amylovora amsB* and *hrpN*, obtained with the reference genes recommended, are in agreement with the results of the whole-transcriptome analysis^37^. The *amsB* gene was more up-regulated in a susceptible apple cultivar Idared than in the resistant one. It is known that amylovoran synthesis is controlled by complex regulatory systems like two component signal transduction systems (TCSTs) which sense environmental signals and induce virulence genes, so possibly in the Free Redstar environment is less favourable for bacteria^8^. Based on the comprehensive data of ranking values obtained by RefFinder, the fourth candidate, *pykA*, could also be used. However, *pykA* had the same GM as *ffh*, but its addition would not make a significant difference in the calculation of the gene expression of *amsB* and *hrpN* genes. Therefore, there was no need to increase the number of reference genes for normalization. The use of multiple reference genes is a more time-consuming and costly approach and is also impractical when a limited amount of RNA is available^50, 51^.

Our additional research of the candidate reference genes indicate that *gyrA* gene was classified as more stably expressed when only bacterial RNA *in planta* were analysed (data not shown). However, in the analysis where *in planta* transcriptomes were compared to the transcriptomes of a pure bacterial culture, *glyA* and *rpoB* were the least stably expressed genes out of the candidate set. This phenomenon was observed by many authors working on gene normalization for RT-qPCR, for example^12^, who obtained a slightly different gene ranking when leaves of *Actinidia deliciosa* were inoculated with low or high doses of *Pseudomonas syringae* pv. *actinidiae* inoculum, or Minervini *et al*.^52^, who found that in stem cell experiments, even minor differences in culture conditions influenced the expression of reference genes. Therefore, the testing of the stability of a set of reference genes in a reaction with all RNAs that will be included in the gene expression analysis is recommended. Additionally, as stated by Petriccione *et al*.^12^, it should be considered that the ideal reference genes can vary with the pathosystem under investigation; therefore, these genes should be carefully selected for each study to conform to the MIQE guidelines. As the candidate reference genes presented in our study showed high and similar efficiency of amplification, the data obtained here could be easily analysed with RefFinder, a tool which does not take into account the efficiency of primers and does not require the transformation of Cq into relative quantities (RQ) to give a comprehensive ranking of genes. However, since the original programs are not time consuming and are easy to use, we recommend using all of the original software packages, which is especially necessary when primer efficiency is not ideal, as these programs can provide additional information such as the optimal number of genes for accurate data normalization.

## Material and Methods

### Bacterial strain and inoculation methods

One-year-old, potted apple trees of a susceptible cultivar, Idared/M.26, and a resistant cultivar, Free Redstar/M.26, were inoculated with *E. amylovora* strain 650 in greenhouse conditions in the spring. The plant shoots were inoculated according to the method described by Kałużna *et al*.^22^. Briefly, trichomes were removed from the surface of the shoots, and the shoots were punctured with a needle on approximately 7 cm of their length from the tip and covered with several 10 µl droplets of bacterial water suspension (~10^9^ cfu/ml). Before inoculation, plants were kept for two days without watering under low humidity conditions. After inoculation, the infected plants were covered for 24 h with a plastic bag to maintain high-humidity conditions and were then kept at a temperature optimal for symptom development (26 °C). Infected shoots were collected at 24 h and 6 days after inoculation. Three independent biological replicates were performed for each sample (12 in total).

### RNA isolation

Total RNA was isolated (from bacterial pellet) from at least 3 shoots of each apple cultivar at each time point, immediately after cutting the shoots from the plant according to the procedure described by Kałużna and coworkers^22^. Additionally, RNA was isolated from a pure culture of *E. amylovora* 650 grown overnight in TY (Bacto Tryptone 0.5%, Yeast Extract 0.3%, CaCl_2_ 0.065%) medium, the same bacteria and growth method used for inoculation purposes. The integrity measurements were performed according to the procedure described by Kałużna and coworkers^22^.

### Selection of candidate reference genes and primer design

Based on a literature screening of commonly used genes for this purpose in other bacteria species^37, 42, 43, 48, 49, 53^, eight candidate reference genes, *ffh* (signal recognition particle protein), *glyA* (serine hydroxymethyltransferase), *gyrA* (DNA gyrase A), *proC* (pyrroline-5-carboxylate reductase), *pykA*, (pyruvate kinase), *recA* (recombinase A), *rpoB* (DNA-directed RNA polymerase subunit beta), *gyrB* (DNA gyrase B), and *rpoC* (DNA-directed RNA polymerase subunit beta), were selected for the analysis of their utility as internal controls for the study of the expression of virulence genes in *Erwinia amylovora*. Based on the gene sequences of *E. amylovora* CFBP 1430 genome (FN434113), primers were designed with the PrimerSelect program of the LASERGENE package (DNASTAR). Selected primers were synthesised by Genomed S.A. (Warszawa, Poland). The specificity and usefulness of the primers were verified twice. First, verification was obtained by specific amplification using PCR, real time PCR and the presence of a single reaction product of the expected size in a 2% agarose gel after electrophoresis, staining with ethidium bromide and visualization under UV light (Figure S1). Second, verification was obtained by real-time PCR, followed by a melting curve analysis of the synthetized product for the verification of the specificity of amplification as indicated by the presence of single melting curve point (Supplementary Figure S2). Of the original candidates, seven reference genes were selected for further study of the stability of gene expression and ranking of the reference genes based on the results obtained from both PCR reactions (Table 1).

### Reverse transcription, quantitative real-time PCR (qPCR) and determination of PCR efficiency

cDNA was synthesized from 200 ng/µl of RNA using an iScript cDNA synthesis kit (Biorad, Hercules, CA). Quantitative real-time PCR was conducted in a Bio-Rad CFX96 thermocycler with SsoAdvanced SYBR Green Supermix (Bio-Rad, Hercules, CA). The reaction mixture, which was 20 μl in total volume, contained 1x SYBR Green Supermix and 0.5 mM of each forward and reverse primer (Table 1) for each gene in separate reactions and 15 ng of cDNA. No-template reactions were used as negative controls. The PCR program was started from one cycle of denaturation at 98 °C for 130 s, followed by 40 cycles at 95 °C for 10 s and then 60 °C for 15 s, finished by a melting curve analysis for the verification of the specificity of amplification in real-time PCR products and the lack of primer dimers. The progressive denaturation of products was carried out at a rising temperature, starting from 65 °C and continuing to 95 °C, with 0.5 °C increments for 5 s each. The amplification efficiency of each reference gene was determined by the generation of a 5-point standard curve based on a ten-fold dilution series of cDNA samples. The efficiency was calculated from the slope of the standard curve generated for each run in the following equation E = 10^(−1/slope)^, where E = 2 and corresponds to 100% efficiency; high/acceptable amplification efficiency equals 90–110%^45^.

### Expression data and stability for the reference genes

Expression data for the reference genes were obtained as quantification cycle (Cq) values obtained from the RNA of pure bacteria and bacteria *in planta*. To determine the stability of the selected reference genes, 5 different programs and algorithms were adopted and analysed: geNorm^30^, NormFinder^31^, BestKeeper^34^, the delta CT method^32^ and the RefFinder web-based tool^33^. For the accurate determination of reference genes in Genorm and NormFinder, Cq the data had to be transformed into relative quantities (RQ). The Cq values from the replicate analyses performed were converted to RQ using the formula, RQ = E^−ΔCq^, where E = PCR efficiency calculated as described above; ΔCq = min Cq (of each gene) - sample Cq. In contrast, the algorithm used for BestKeeper, the delta CT method and the RefFinder web-based tool used the raw non-transformed Cq data.

The GeNorm algorithm calculates an average expression stability M-value for each gene from a pool of reference genes used in analysis. The M-value is defined as the average pairwise variation in a particular gene with all other potential reference genes. Genes with the lowest M values have the most stable expression^30^. By the exclusion of less stably expressed genes we can select the most stably expressed genes, which can be used for normalization studies. In this software, the minimum number of genes required for normalization can be determined by pairwise variation V_n_/V_n+1_^30^.

NormFinder identifies stably expressed genes among a set of candidate normalization genes based on a mathematical model that enables the estimation of the intra and inter-group variation of the sample set. By combining the results obtained, a stability value (SV) is calculated^31^.

BestKeeper is an Excel-based tool that helps in selection the of the best reference genes after the calculation of variables: Pearson correlation coefficient (*r*), the standard deviation (SD) and a coefficient of variance (CV). Any gene with a SD higher than 1 is treated as inconsistent. For stably expressed genes, the BestKeeper Index is calculated based on the geometric mean of Ct values of reference genes^34^.

The delta Ct approach compares the relative expression of all possible ‘pairs of genes’ within each sample. The stability of the reference genes is ranked according to the repeatability of the gene expression differences among samples^32^.

Finally, the raw Cq values of each gene (without taking into account PCR efficiencies) were used to calculate the comprehensive ranking of reference genes using the web-based tool RefFinder^33^. The program is based on the ranking obtained from each program (geNorm, Normfinder, BestKeeper, and the delta Ct method). It assigns an appropriate weight to an individual gene and calculates the geometric mean (GM) of their weights, giving an overall comprehensive ranking.

### Expression analysis of the target virulence genes *hrpN* and *amsB*

The expression profile analysis of the target genes coding critical virulence factors *amsB*, which is involved in the biosynthesis of amylovoran, and *hrpN* coding harpin, a secreted protein that elicits the hypersensitive response (HR) in non-hosts and is also required for pathogenicity in host plants, was carried out with the reference genes indicated by the mathematic algorithms. Primers for the target genes were designed with the PrimerSelect program of the LASERGENE package (DNASTAR) and were synthesized by Genomed S.A. (Warszawa). The primer pairs for the *amsB* and *hrpN* genes are listed in Table 1. The same conditions and criteria as for the reference genes were used for RT-qPCR. To calculate the relative fold changes in the gene expression of *E. amylovora* strain 650 after its infection of the susceptible cultivar Idared and the resistant cultivar Free Redstar, the data obtained by Cfx96 (Bio-rad) was analysed using the comparative 2^−ΔΔCt^ method and normalized to the selected reference genes^17^. Three-way ANOVA was used for the relative expression gene data with gene, apple cultivare, time as a factors. Newman-Keuls test, was performed to determine significance of differences between means.

## Electronic supplementary material

Supplementary information
