# Supplementary material for: Validation of reference genes for the normalization of the RT-qPCR gene expression of virulence genes of Erwinia amylovora in apple shoots
Source: Sci Rep. 2017 May 17;7:2034. doi: 10.1038/s41598-017-02078-4 (PMC5435713; doi:10.1038/s41598-017-02078-4)

Supplementary information

Validation of reference genes for the normalization of the RT-qPCR gene expression of virulence genes of *Erwinia amylovora* in apple shoots

Monika Kałużna*, Anita Kuras & Joanna Puławska

Research Institute of Horticulture, Konstytucji 3 Maja 1/3, 96-100 Skierniewice, Poland

*To whom correspondence should be addressed: Research Institute of Horticulture, Department of Phytopathology, Pomologiczna 18, 96-100 Skierniewice; E-mail: monika.kaluzna@inhort.pl

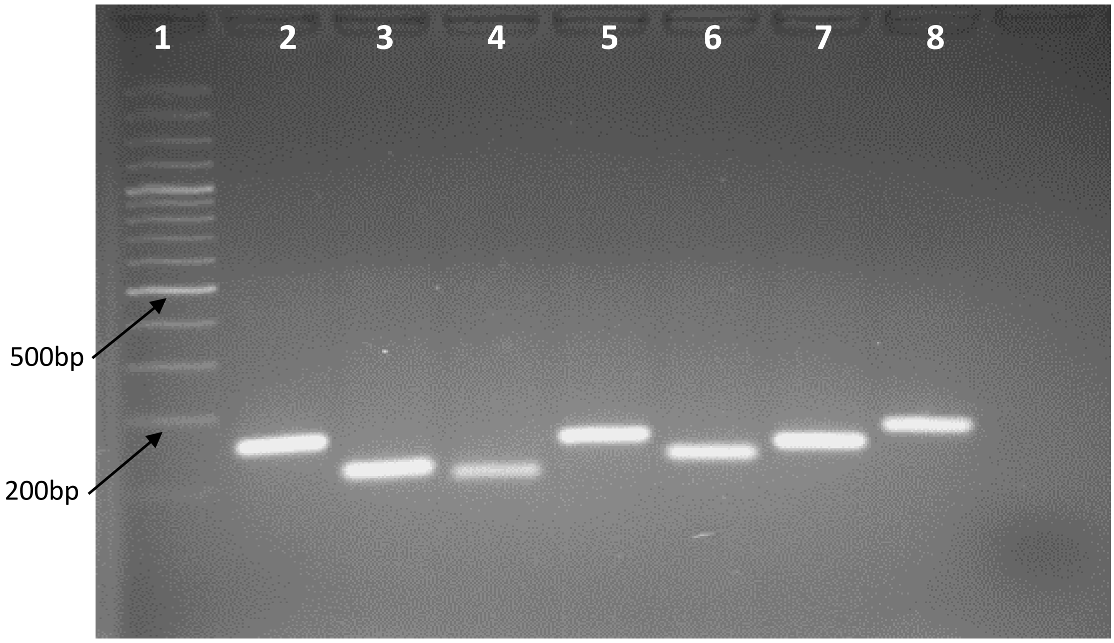

Figure S1. Agarose gel electrophoresis showing specific real-time PCR products of the expected size obtained for each candidate reference gene with designed primers. 1- O'GeneRuler 100-3000bp (#SM1153, Thermo Scientifc, Lithuania), 2-*ffh*, 3-*glyA*, 4-*gyrA*, 5-*proC*, 6-*pykA*, 7-*recA*, 8-*rpoB*

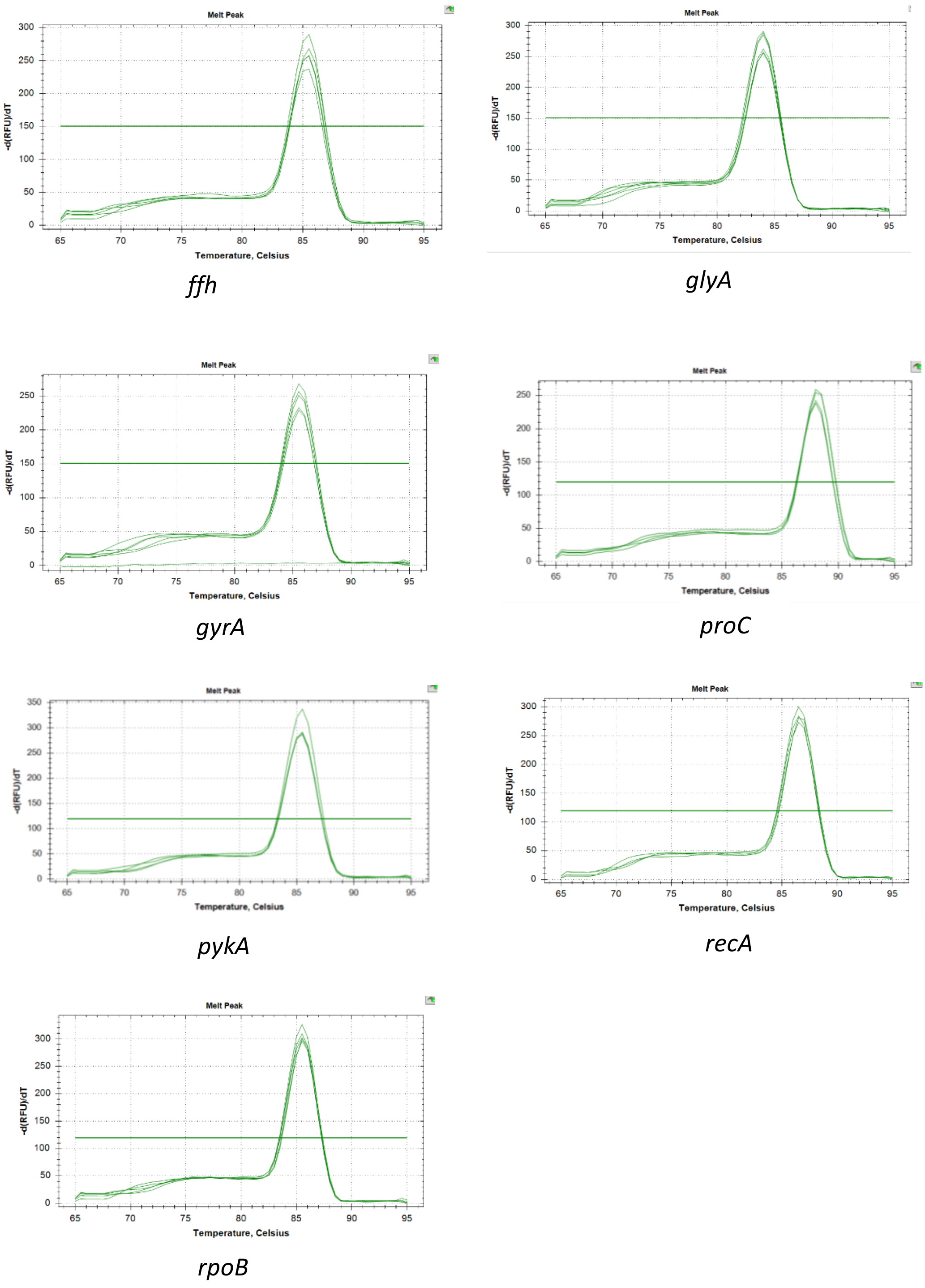

Figure S2.. Melting curves of amplification products of each candidate reference genes with single peak obtained.

Figure S3A. RefFinder output for all samples, comprehensive ranking.

Comprehensive ranking of all analyzed samples from Idared and Free Redstar generated by RefFinder web-based comprehensive tool. Table presents the stability values obtained and a graph with most stable to the least stable genes.

Genes	Geomean of ranking values
proC	1.41
recA	2.06
ffh	2.63
pykA	2.63
gyrA	5.23
rpoB	5.73
glyA	7.00

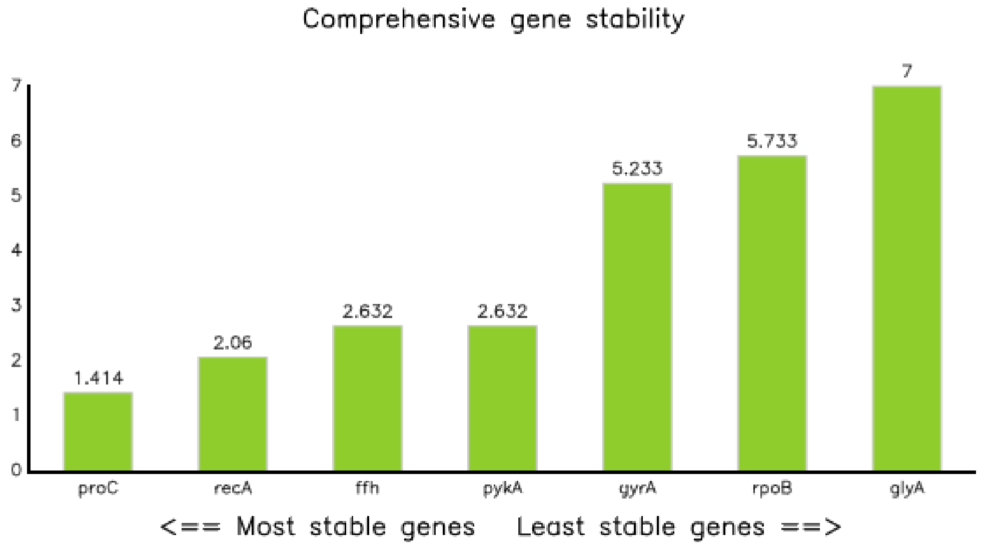

Figure S3B. RefFinder output for all samples, geNorm ranking.

geNorm ranking of all analyzed samples from Idared and Free Redstar generated by RefFinder web-based comprehensive tool. Table presents the stability values and a graph with most stable to the least stable genes.

Gene name	Stability value
proC | recA	0.304
ffh	0.367
pykA	0.408
rpoB	0.519
gyrA	0.563
glyA	0.616

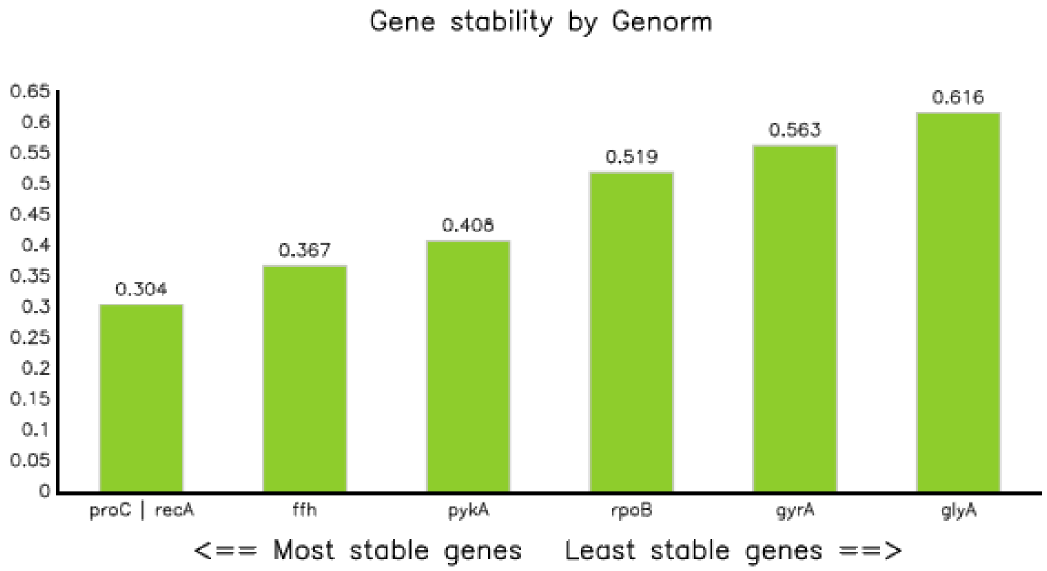

Figure S3C. RefFinder output for all samples, NormFinder ranking.

NormFinder ranking of all analyzed samples from Idared and Free Redstar generated by RefFinder web-based comprehensive tool. Table presents the stability values and a graph with most stable to the least stable genes.

Gene name	Stability value
proC	0.345
pykA	0.360
recA	0.368
ffh	0.433
gyrA	0.447
rpoB	0.501
glyA	0.633

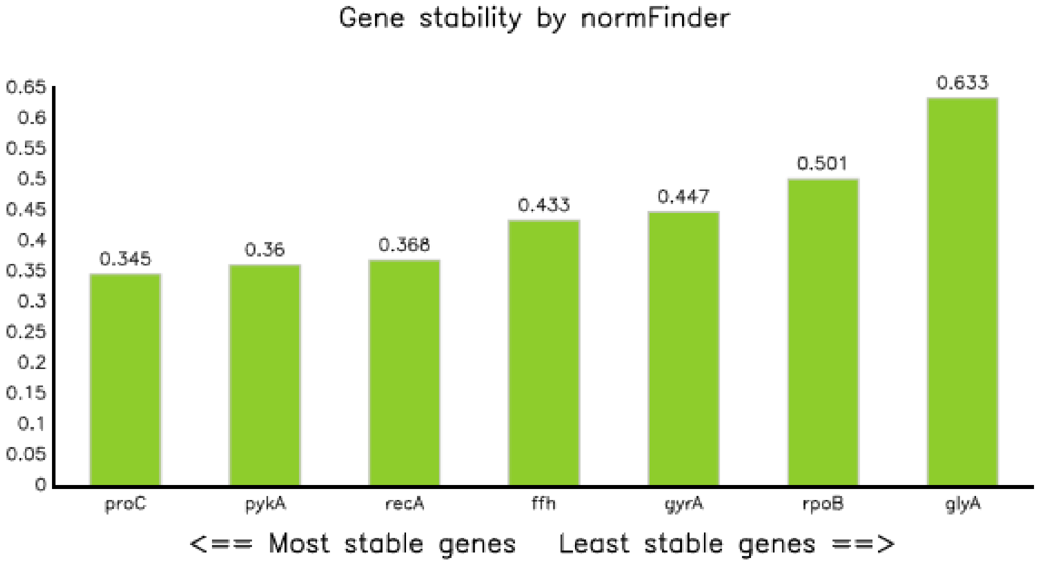

Figure S3D. RefFinder output for all samples, BestKeeper ranking

BestKeeper ranking of all analyzed samples from Idared and Free Redstar generated by RefFinder web-based comprehensive tool. Tables presents the output data generated from BestKeeper program. Below the graph with most stable to the least stable genes.

CP data of housekeeping Genes by BEST KEEPER

	ffh	*glyA*	*gyrA*	*proC*	*pykA*	*recA*	*rpoB*
n	14	14	14	14	14	14	14
geo Mean [CP]	21.48	20.26	21.82	22.60	22.41	19.83	19.84
AR Mean [CP]	21.49	20.28	21.83	22.61	22.42	19.84	19.85
min [CP]	20.22	18.64	20.50	21.68	21.36	18.56	18.36
max [CP]	22.42	21.27	22.71	24.18	23.71	21.36	20.99
**std dev [+/- CP]**	**0.46**	**0.67**	**0.57**	**0.50**	**0.48**	**0.49**	**0.59**
CV [% CP]	2.14	3.29	2.61	2.22	2.16	2.49	2.95
min [x-fold]	-2.40	-3.08	-2.49	-1.89	-2.08	-2.40	-2.78
max [x-fold]	1.91	2.01	1.86	3.00	2.45	2.90	2.23
std dev [+/- x-fold]	1.37	1.59	1.48	1.42	1.40	1.41	1.50

Pearson correlation coefficient (r) by BEST KEEPER

	ffh	*glyA*	*gyrA*	*proC*	*pykA*	*recA*	*rpoB*
glyA	0.373	-	-	-	-	-	-
p-value	0.190	-	-	-	-	-	-
gyrA	0.340	0.787	-	-	-	-	-
p-value	0.235	0.001	-	-	-	-	-
proC	0.761	0.414	0.504	-	-	-	-
p-value	0.002	0.141	0.066	-	-	-	-
pykA	0.602	0.400	0.472	0.867	-	-	-
p-value	0.023	0.157	0.088	0.001	-	-	-
recA	0.856	0.419	0.486	0.900	0.743	-	-
p-value	0.001	0.136	0.078	0.001	0.002	-	-
rpoB	0.415	0.539	0.820	0.538	0.668	0.520	-
p-value	0.140	0.046	0.001	0.047	0.009	0.057	-

Pearson correlation coefficient (r)

BestKeeper vs.	*ffh*	*glyA*	*gyrA*	*proC*	*pykA*	*recA*	*rpoB*
coeff. of corr. [r]	0.744	0.728	0.811	0.860	0.825	0.852	0.814
p-value	0.002	0.003	0.001	0.001	0.001	0.001	0.001

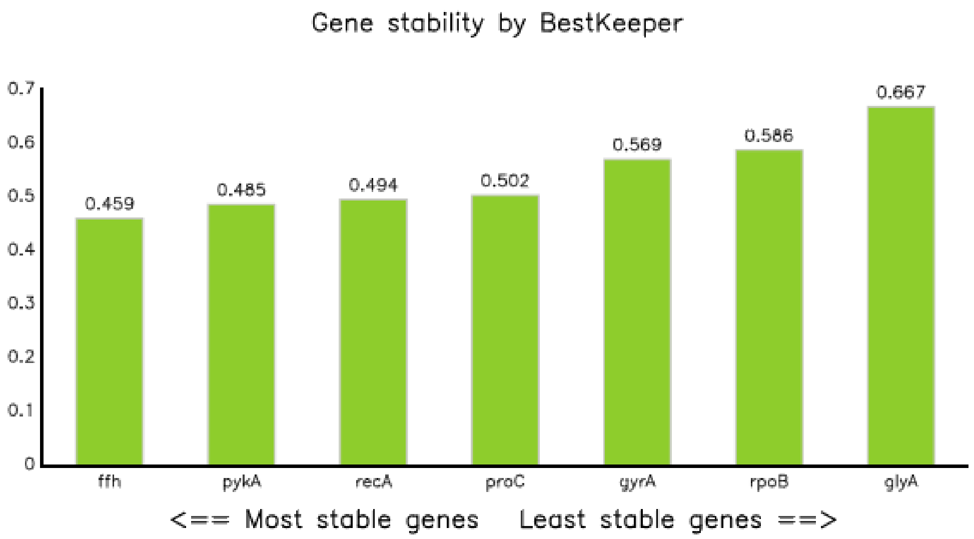

Gene stability by BestKeeper
0.7
0.6
0.5
0.4
0.3
0.2
0.1
0
0.459
0.485
0.494
0.502
0.569
0.586
0.667
ffh
pykA
recA
proC
gyrA
rpoB
glyA
<== Most stable genes   Least stable genes ==>

Figure S3E. RefFinder output for all samples, Delta Ct ranking

Delta Ct ranking of all analyzed samples from Idared and Free Redstar generated by RefFinder web-based comprehensive tool. Table presents the stability values and a graph with most stable to the least stable genes.

Genes	Average of STDEV
proC	0.55
recA	0.56
pykA	0.57
ffh	0.60
gyrA	0.63
rpoB	0.66
glyA	0.75

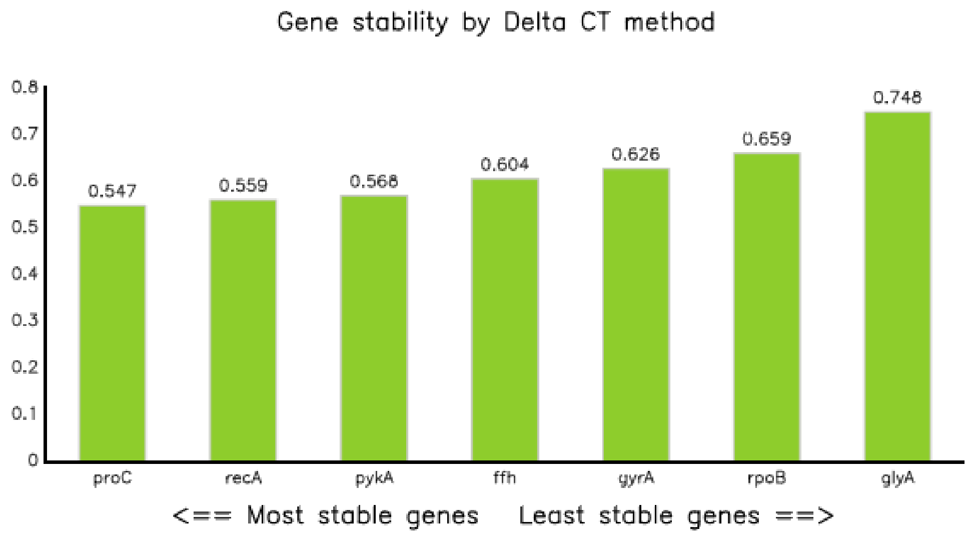

Supplement: Supplementary file 1 — Supplementary information [file 41598_2017_2078_MOESM1_ESM.pdf]
